# Beta‐propeller protein‐associated neurodegeneration: a case report and review of the literature

**DOI:** 10.1002/ccr3.1358

**Published:** 2018-01-04

**Authors:** Kjersti Eline Stige, Ivar Otto Gjerde, Gunnar Houge, Per Morten Knappskog, Charalampos Tzoulis

**Affiliations:** ^1^ Department of Neurology Haukeland University Hospital Bergen Norway; ^2^ Department of Clinical Medicine University of Bergen Bergen Norway; ^3^ Center for Medical Genetics and Molecular Medicine Haukeland University Hospital Bergen Norway; ^4^ Department of Clinical Science K.G. Jebsen Centre for Neuropsychiatric Disorders University of Bergen Bergen Norway

**Keywords:** Beta‐propeller protein‐associated neurodegeneration, neurodegeneration with brain iron accumulation, neurodegeneration with iron accumulation, *WDR45*

## Abstract

Beta‐propeller protein‐associated neurodegeneration (BPAN) is a rare disorder, which is increasingly recognized thanks to next‐generation sequencing. Due to a highly variable phenotype, patients may present to pediatrics, neurology, psychiatry, or internal medicine. It is therefore essential that physicians of different specialties are familiar with this severe and debilitating condition.

## Introduction

Beta‐propeller protein‐associated neurodegeneration (BPAN) was first described by Haack et al. in 2012 1. Before the elucidation of its genetic etiology, the disorder was termed static encephalopathy of childhood with neurodegeneration in adulthood (SENDA). This term reflected the disorder′s typical clinical course, comprising static psychomotor retardation in childhood followed by progressive deterioration in adolescence or young adulthood with progressive dystonia, parkinsonism, and dementia. After the discovery of pathogenic mutations in the *WDR45* gene, the disorder was renamed BPAN in keeping with the naming conventions of other forms of neurodegeneration with brain iron accumulation (NBIA).

Beta‐propeller protein‐associated neurodegeneration is a rare disease. NBIA disorders have a prevalence of 1/1,000,000, and of these, BPAN constitutes only 7% 2. Since 2012, 68 cases of BPAN have been reported in PubMed, but systematic epidemiological studies are lacking and the true incidence and prevalence remain therefore unknown. The aim of this article was to provide an overview of BPAN focusing on clinical history, genetics, and pathophysiology. Patient management will not be discussed in detail as this was recently published elsewhere 3.

## Methods

### Case report

To illustrate the typical history and clinical phenotype of BPAN, we present a case that was recently diagnosed at our hospital. This patient is, to our knowledge, the first published case of BPAN in Scandinavia.

### Literature review

A systematic PubMed search was performed until 23 June 2017 using the following keywords: “BPAN,” “beta‐propeller protein‐associated neurodegeneration,” and “*WDR45*”. A total of 42 articles were retrieved and evaluated by the authors. A total of 34 articles contained information on case reports, of which five were excluded because they were not available in the English language (*n* = 1), provided limited clinical information (*n* = 2), or were not possible to obtain (*n* = 2).

## Case Report

**Figure 1:**
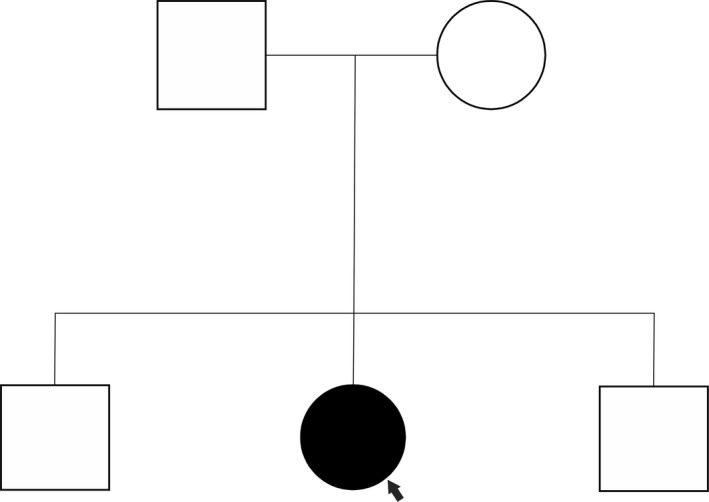
The pedigree structure of the Norwegian family; the affected individual is shown as a filled symbol and is marked with an arrow.

A 33‐year‐old woman of Norwegian descent was referred for neurological evaluation due to rapid motor deterioration. Her parents and two brothers were healthy (Fig. 1). She had been born after a normal pregnancy and delivery and had an uncomplicated neonatal and early development until the age of 6 months, when she was admitted due to episodic cyanosis and eye‐rolling/deviation raising suspicion about epileptic seizures. The parents reported persistent crying and intermittent fever during the weeks prior to admittance. Electroencephalography (EEG) showed high‐voltage activity with bursts of spike and wave activity, similar to that seen in children with infantile spasms. Suspicion of viral encephalitis was raised, but CSF examination was unremarkable. After discharge, the patient continued having complex partial seizures, characterized by automatisms in the form of lip‐smacking and swallowing, which later converted to short pure consciousness lapses, similar to absence seizures. Interictal EEG continued to show epileptic activity of variable localization. Computed tomography (CT) of the brain showed generalized cerebral atrophy.

Her early motor development was normal. She walked at 13 months and was able to climb as a child. At 18 months, it was noted that her language and cognitive development were delayed. At 2.5 years of age, she lost her speech with the exception of a few words and communicated mostly by inarticulate sounds. Intermittent strabismus was described during her school years, and occlusion therapy was attempted but was unsuccessful due to poor compliance. Her hearing developed normally. She was treated with salivary gland surgery at the age of seven due to drooling. Concentration difficulties, anxiety, and phobias were also reported.

The patient′s epilepsy responded to treatment with phenobarbital, but due to side effects, she was switched to carbamazepine and finally valproic acid, which was well tolerated and provided optimal long‐term seizure control. Her antiepileptic therapy was discontinued at the age of 12, as she had become seizure free. At the age of 17, she developed myoclonic jerks, particularly in her upper extremities, which improved after initiation of clonazepam. Magnetic resonance imaging (MRI) showed signs of metal accumulation in the basal ganglia with conspicuously low T2 signal in the *basal ganglia*. Susceptibility‐weighted imaging (SWI) was not performed.

**Figure 2:**
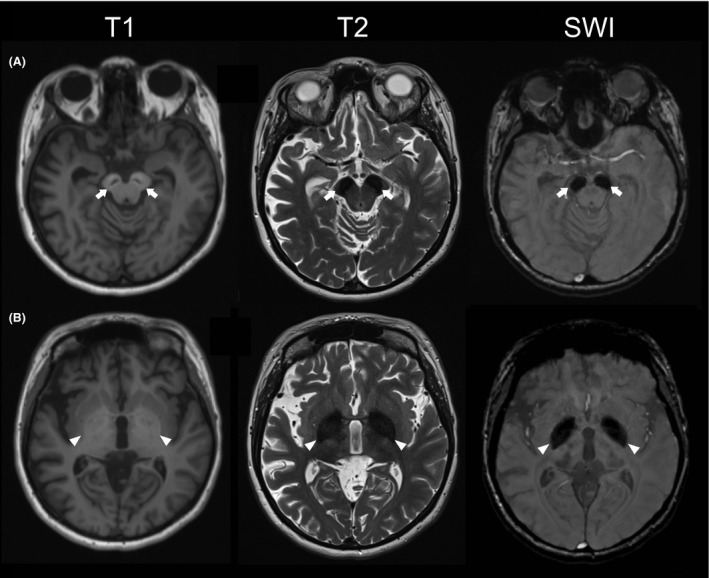
MRI findings in BPAN. Brain MRI of the Norwegian patient at the age of 33 years, showing typical findings for BPAN. (A) Axial T1‐weighted image at the level of the midbrain shows symmetric hyperintense “halos” surrounding a band of central hypointensity in the *substantia nigra* (arrows). Axial T2‐weighted and SWI image of the same area shows prominent hypointensities in the *substantia nigra* and cerebral peduncles (arrows). (B) Axial images at the level of the striatum show high T1 signal and low T2 and SWI signal in the *globus pallidus*. The low SWI signal corresponds to areas of increased iron deposition (arrowheads).

At the age of 32, her motor function began deteriorating. She developed flexion of her left elbow, wrist, and fingers. This was followed by rapid worsening of general locomotion and gait with frequent falls. Her cognitive and language skills also declined further, and she developed urinary and fecal incontinence. On clinical examination, she was obese, short in stature, but with a generally “happy demeanor” and had severe intellectual disability and poor language skills. She had pronounced dystonic posturing and rigidity in all extremities, bradykinesia, freezing of gait, and stooped posture. Tendon reflexes were normal. She communicated mainly through inarticulate sounds and gestures. Dysmorphic features were noted in the form of short distal phalanx of the thumbs, enlarged gap between the upper teeth, and strabismus. MRI showed a hyperintense “halo” surrounding a band of central hypointensity in the *substantia nigra* and cerebral peduncles on T1‐weighted images and hypointensity in the *substantia nigra* and *globus pallidus* (mostly pronounced in *substantia nigra*) on T2‐weighted images. SWI showed prominent hypointensity consistent with metal deposition in the areas mentioned above. There was also marked cerebral atrophy and slight atrophy of vermis (Fig. 2).

Whole‐exome sequencing (WES) was performed using the SeqCap EZ MedExome Target Enrichment System (Roche) and Illumina NextSeq 500 platform with NextSeq 500/550 High Output v2 kit (2 × 75 cycles). Data analysis was performed as previously described 4. Variant filtering was carried out in the NGS module of Cartagenia. WES identified a heterozygous frameshift mutation in the *WDR45* gene: c.1007_1008del, p.Y336Cfs*5. This mutation has been previously reported in two patients with BPAN and is known to be pathogenic. The patient's parents tested negative for the mutation consistent with a de novo origin. Based on these findings, the patient was diagnosed with BPAN. Treatment with levodopa/carbidopa (100/25 mg qid) led to substantial improvement in motor function (in particular, improvement of the bradykinesia), language skills (slight improvement of vocabulary), and her general condition.

## Literature Review

### Demographics and clinical spectrum of BPAN

A total of 64 patients were included in the analysis, comprising 55 women (85.9%) and nine men (14.1%) (Tables 1 and 2) 1, 5, 6, 7, 8, 9, 10, 11, 12, 13, 14, 15, 16, 17, 18, 19, 20, 21, 22, 23, 24, 25, 26, 27, 28, 29, 30, 31, 32. Mean age at diagnosis was 27.6 ± 14 years (range 1–52), and mean age at deterioration was 27.2 ± 5.7 (range 13–39). The origin of the mutations was assessed in 47 patients and found to be de novo in 43 (91.5%) and inherited in four (8.5%).

**Table 1:** Summary of reported cases with BPAN

PN	Published in	S	cDNA	Protein	I	Diagnosis age	RLF	Epilepsy	Deterioration age	Parkinsonism	Dystonia	Dysmorphia	PCD	L‐dopa response	L‐dopa dyskinesia
1	Nishioka ‐15	F	c.969_970insT	p.V324Cfs*18	dn	30	−	+	29	+	+	na	+	+	+
2	Nishioka ‐15	F	c.585_588delTA	p.I196Sfs*26	dn	37	−	+	30	+	+	na	+	+	+
3	Nishioka ‐15	F	c.414_419delGTTGA	p.E138_F139del	dn	36	−	+	32	+	−	na	+	+	+
4	Nishioka ‐15	F	c.628T>C	p.S210P	na	33	−	+	32	+	−	na	+	+	−
5	Nishioka ‐15	F	c.400C>T	p.R134*	na	35	−	+	34	+	−	na	+	+	−
6	Nishioka ‐15	F	c.587_588delTA	p.I196Sfs*26	dn	33	−	−	28	+	+	na	+	+	+
7	Nishioka ‐15	F	c.293T>C	p.L98P	dn	41	−	+	39	+	−	na	+	+	−
8	Hayflick ‐13, Haack ‐12	F	c.1007_1008del	p.Y336Cfs*5	dn	23	+	+	na	+	+	na	+	na	na
9	Hayflick ‐13, Haack ‐12	F	c.38G>C	p.R13P	dn	44	−	−	26	+	+	na	+	+	+
10	Hayflick ‐13, Haack ‐12	F	c.‐1_5del	p.M1?	na	30	+	+	26	+	+	na	+	+	+
11	Hayflick ‐13, Haack ‐12	F	c.293T>C	p.L98P	na	29	−	+	na	−	+	na	+	na	na
12	Hayflick ‐13, Haack ‐12	F	c.476del	p.L159Rfs*2	na	43	−	−	26	+	+	na	+	na	na
13	Hayflick ‐13, Haack ‐12	F	c.19C>T	p.R7*	na	34	−	−	25	+	+	na	+	+	+
14	Hayflick ‐13, Haack ‐12	F	c.56‐1G>A	Splicing defect	na	22	+	+	15	+	+	na	+	na	na
15	Hayflick ‐13, Haack ‐12	F	c.700C>T	p.R234*	na	39	−	+	29	+	+	na	+	+	+
16	Hayflick ‐13, Haack ‐12	F	c.400C>T	p.R134*	dn	49	−	+	37	+	+	na	+	+	+
17	Hayflick ‐13, Haack ‐12	M	c.228_229del	p.E76Dfs*38	dn	37	−	−	27	+	+	na	+	+	+
18	Hayflick ‐13, Haack ‐12	F	c.405_409del	p.K135Nfs*2	na	40	−	−	30	+	+	na	+	+	+
19	Hayflick ‐13, Haack ‐12	F	c.359dup	p.K121Efs*18	dn	45	−	−	31	+	+	na	+	na	na
20	Hayflick ‐13, Haack ‐12	F	c.830 + 1G>A	Splicing defect	dn	37	+	−	26	+	+	na	+	na	na
21	Hayflick ‐13, Haack ‐12	M	c.19dup	p.R7Pfs*64	na	31	−	+	28	+	+	na	+	na	na
22	Hayflick ‐13, Haack ‐12	F	c.235 + 1G>A	Splicing defect	dn	35	−	−	30	+	+	na	+	na	na
23	Hayflick ‐13, Haack ‐12	F	c.1007_1008del	p.Y336Cfs*5	dn	24	−	+	19	+	+	na	+	na	na
24	Hayflick ‐13, Haack ‐12, Paudel ‐15	F	c.694_703del	p.L232Afs*53	dn	44	−	−	29	+	+	na	+	+	+
25	Hayflick ‐13, Haack ‐12	F	c.183C>A	p.N61K	dn	17	−	+	16	+	+	na	+	+	+
26	Hayflick ‐13, Haack ‐12	M	c.1025_1034del insACATATTT	p.G342Dfs*12	na	31	−	+	26	+	+	na	+	+	+
27	Hayflick ‐13, Haack ‐12	F	c.55 + 1G>C	Splicing defect	dn	43	+	+	25	+	+	na	+	+	+
28	Hayflick ‐13	F	c.830 + 2T>C	Splicing defect	dn	27	−	−	20	+	+	na	+	na	na
29	Hayflick ‐13	F	c.1A>G	Start codon abolished	na	31	+	+	29	−	+	na	+	na	na
30	Hayflick ‐13	F	c.186delT	p.L63Wfs*19	na	16	+	+	15	na	+	na	+	na	na
31	Hoffjan ‐16	F	c.440‐2A>G	Splicing defect	dn	5	+	+	−	−	−	+	−	na	na
32	Verhoeven ‐14	F	c.622_663del	p.F221*	dn	33	na	na	32	−	+	na	+	na	na
33	Verhoeven ‐14	F	c.752_754del	p.S251del	na	52	na	+	35	**+**	+	−	+	−	na
34	Verhoeven ‐14	F	c.1030del	p.C344fs	dn	42	na	−	33	na	+	−	+	+	−
35	Xixis ‐15	F	c.400C>T	p.R134*	dn	6	na	+	−	−	−	na	−	na	na
36	Saitsu ‐13	F	c.439 + 1G>T	p.G147V;V147_L148ins8	dn	33	na	+	26	**+**	+	na	+	na	na
37	Saitsu ‐13	F	c.516G>C	p.D174Vfs*29	dn	28	na	+	25	**+**	+	na	+	na	na
38	Saitsu ‐13	F	c.437dupA	p.L148Afs*3	na	40	na	+	30	na	+	na	+	na	na
39	Saitsu ‐13	F	c.637C>T	p.Q213*	na	51	na	−	24	na	+	na	+	na	na
40	Saitsu ‐13	F	c.1033_1034dupAA	p.N345Kfs*67	dn	33	na	+	23	**+**	+	na	+	na	na
41	Takano ‐16, Morikawa ‐17	F	c.813‐1G>C	Splicing defect	dn	3	−	+	−	−	−	+	−	na	na
42	Wynn ‐17	F	c.597_598delGT	p.L201Kfs*21	dn	34	na	−	32	−	+	na	na	na	na
43	Yoganathan ‐16	F	c.400G>A	p.R134Ter	dn	5	**+**	+	−	−	−	−	−	na	na
44	Long ‐15	F	c.251A>G	p.D84G	dn	18	−	−	−	−	−	−	−	na	na
45	Zarate ‐16	M	c.161_163delTGG	p.V54del	inh	20	−	na	na	na	na	+	na	na	na
46	Zarate ‐16	F	c.161_163delTGG	p.V54del	inh	14	−	−	−	na	na	+	−	na	na
47	Tschentscher ‐15 + Hattingen ‐17	F	c.626C>A	p.A209D	dn	33	na	+	27	+	na	na	na	na	na
48	Van Goethem ‐14	F	c.488delC	p.P163Rfs*34	dn	na	na	+	22	−	+	na	+	na	na
49	Okamoto ‐14	F	c.C868T	p.Q290*	dn	6	+	+	−	−	−	+	−	na	na
50	Ichinose ‐14	F	c.519 + 1_519 + 3del	na	dn	31	na	−	30	+	+	na	na	+	na
51	Ohba ‐14	F	c.830 + 1G>A	p.L278*	dn	14	+	+	−	−	+	+	−	na	na
52	Ozawa ‐14	F	c.322del	p.S108Lfs*10	dn	39	−	+	28	−	+	na	+	na	na
53	Khalifa ‐15	F	c.587‐588del	p.196fs	dn	11	+	−	−	−	−	+	−	na	na
54	Rathore ‐14	F	c.342‐2A>C	na	dn	15	−	+	13	−	+	na	+	+	na
55	Abidi ‐16	M	Deletion of *WDR45*	na	dn	na	−	+	−	−	+	na	−	na	na
56	Ryu ‐15	F	c.345‐1G>A	r.345_439del	na	43	−	−	na	+	na	na	na	na	na
57	Spiegel ‐16	M	c.1007_1008delAT	p.Y336C*5	dn	na	−	−	−	−	−	na	−	na	na
58	Nakashima ‐16	M	c.131‐1G>A	na	dn	1	−	+	−	−	−	na	−	na	na
59	Nakashima ‐16	M	c.248G>A	p.W83*	dn	2	−	+	−	−	−	na	−	na	na
60	Nakashima ‐16	M	c.400C>T	p.R134*	inh	7	−	+	−	−	−	−	−	na	na
61	Nakashima ‐16	F	c.400C>T	p.R134*	inh	7	na	+	−	−	−	+	−	na	na
62	Arauju ‐17	F	c.447_448del	p.C149*	dn	8	**+**	+	na	−	+	+	+	na	na
63	Arauju ‐17	F	c.447_448del	p.C149*	dn	8	**+**	**+**	na	−	+	+	+	na	na
64	Our case	F	c.1007_1008del	p.Y336Cfs*5	dn	33	−	+	32	+	+	+	+	+	−

PN, patient number; S, sex; I, inheritance; RLF, Rett‐like features; PCD, progressive cognitive decline; F, female; M, male; na, not available; dn, de novo; inh, inherited; +, yes; −, no.

**Table 2:** Summary of clinical features

Feature	*n*	%
Female	55/64	85.9
Male	9/64	14.1
De novo mutation	43/47	91.5
Inherited mutation	4/47	8.5
Rett‐like features	14/50	28
Epileptic seizures	42/62	67.7
Parkinsonism	35/58	60.3
Dystonia	44/60	73.3
Dysmorphic features	11/16	68.8
Progressive cognitive decline	44/59	74.6
L‐dopa response	22/23	95.7
L‐dopa‐induced dyskinesias	15/20	75

*n*: number of patients with the feature/total evaluated.

All patients had delayed psychomotor development and intellectual disability manifesting from infancy or early childhood characterized by pronounced loss of expressive language skills. In addition, 44 of 59 patients (74.6%) developed progressive cognitive decline upon reaching adolescence or early adulthood. The majority had epileptic seizures (42/62, 67.7%) and movement disorders including dystonia (44/60, 73.3%) and parkinsonism (35/58, 60.3%). Epileptic seizures started in early childhood and showed a spectrum ranging from focal to generalized seizures and epileptic spasms. Multiple seizure types were commonly seen in the same individuals 6, 16, 20. The epilepsy was generally most severe in childhood and improved with advancing age 3. Dystonia and parkinsonism developed in adolescence or early adulthood when the motor function started deteriorating and caused severe motor disability with many patients becoming wheelchair‐dependent or bedridden.

Rett‐like features, including developmental regression, loss of purposeful hand skills, stereotypic hand movements, and bruxism, were seen in 14 patients (14/50, 28%). All but one of these 23 had atypical Rett syndrome or Rett‐like features, meaning that they did not fulfill all formal diagnostic criteria for Rett syndrome.

Spasticity, sleep disturbances, and ocular/visual defects have been variably reported to be a part of the phenotype. A systematic statistical evaluation of these was not possible due to inconsistent reporting and incomplete descriptions in the literature. Limb spasticity of highly variable severity has been described in both children and adults. A wide range of sleep disturbances has been reported including REM sleep disorder 18, excessive movement during sleep 18, circadian rhythm sleep disorder 18, 24, hypersomnolence with choreiform movements at onset of sleep 18, parasomnia with nocturnal screaming 18, and unspecified sleep disorders 5, 18, 22. Ocular/visual involvement has been reported in 17 patients and comprises myopia 7, 18, 22, astigmatism 18, strabismus 7, 26, abnormal pupillary shade 18, spontaneous retinal detachment 18, bilateral partial retinal coloboma 18, patchy loss of pupillary ruff 18, difficulties with eyesight with intermittent double‐vision 18, bilateral optic disk pallor 24, bilateral optic atrophy 15, 24, increased visual evoked potential (VEP) latency 18, cortical blindness 16, and retinitis pigmentosa 6.

Dysmorphic features have not been systematically characterized in BPAN. Definite data were only available for 16 of 64 patients (25%) of whom 11 (68.8%) were reported to have dysmorphic features. These included microcephaly 25, abnormal nasal bridge (depressed, high, wide, and flat) 9, 26, 27, 30, a small mouth 26, tented upper lip 26, hypertelorism 9, 26, epicanthal folds 9, 26, downslanting palpebral fissures 9, 27, large ears 26, 27, bilateral low‐set ears 9, low hanging columella 27, short philtrum 9, 30, high palate 9, downturned mouth and micrognathia 9, narrow face 30, narrow nose 30, thin upper lip 30, kyphosis 23, flat and almost rocker bottom feet 7, fingers tapered 7, 27 with fifth finger clinodactyly 7, partial synophrys 7, and congenital *talipes varus* 6. Small cold hands and feet were also reported 23. It was not possible to identify any common dysmorphic features from this analysis.

Other clinical features of BPAN include neuropsychiatric symptoms 18, 22, 24, “happy demeanor” 7, and excessive drooling 7, 25. Chorea has been reported in one patient 12. Bilateral sensory neural hearing loss has also been reported 15 as well as auditory agnosia 24.

### Imaging findings

The vast majority of patients with BPAN (55/61, 90.2%) had MRI findings consistent with iron deposition in the basal ganglia. The two most typical findings were as follows:

- Hypointense signal in the *substantia nigra* and *globus pallidus* on T2‐weighted or iron‐sensitive sequences such as SWI. This finding was more prominent in older individuals. The T2 hypointensity was generally more pronounced in the *substantia nigra* compared to the *globus pallidus*, a feature that may help distinguish BPAN from other forms of NBIA.
- Hyperintense “halo” surrounding a band of central hypointensity in the *substantia nigra* and cerebral peduncles on T1‐weighted images. This finding is generally regarded as pathognomonic for BPAN.

Other features included cerebral atrophy (44/64, 68.8%), cerebellar atrophy (17/64, 26.6%), delayed myelination 6, 9, 15, 26, thin corpus callosum 25, 26, and dilated ventricles 9, 10. Radiological features are summarized in Table 3.

**Table 3:** Summary of radiological features

Radiological findings	*n*	%
Iron deposition	55/61	90.2
Cerebral atrophy	44/63	69.8
Cerebellar atrophy	17/63	27
Delayed myelination	6/63	9.5
Thin corpus callosum	2/63	3.2
Dilated ventricles	2/63	3.2

*n*: number of patients with the feature/total evaluated.

### Treatment

Treatment with oral levodopa leads to clinical improvement in 20 of 21 (95.2%) of BPAN patients with parkinsonism. Positive effects included amelioration of rigidity and bradykinesia, improved affect, appetite, and interest in activities 14, 18, 21, 22. Slight improvement of expressive language was seen in one case 14. The majority of patients (15/20, 75%) also had treatment side effects, however, in the form of motor fluctuation and dyskinesias 18. Clinical improvement was reported in two cases without parkinsonism. One of these experienced amelioration of dystonia, whereas the nature of improvement is not specified in the other. As seen in Table 2, several BPAN patients with parkinsonism (14/35, 40%) were either not treated with levodopa or no information was given on treatment effect.

## Pathophysiology

BPAN is caused by mutations in the *WDR45* gene. *WDR45* encodes WIPI‐4 (WD repeat domain phosphoinositide‐interacting protein 4), which is part of the WD40 repeat protein family. This group of proteins facilitates the assembly of multiprotein complexes and is important in many essential biological processes, such as signal transduction, cell cycle progression, gene regulation, and apoptosis 33, 34. WD40 domain‐containing proteins assume a symmetric, seven‐bladed, beta‐propeller platform structure that supports protein–protein interactions. WIPI‐4 is phylogenetically related to two yeast proteins (ATG18 and ATG21) that are part of a recycling system involved in transferring and recycling components from the isolation membrane (also known as phagophore) to the growing autophagosome 35, 36. Available evidence suggests that human WIPI4 protein is involved in autophagy and interacts with the known autophagy factors ATG2A and ATG2B 3, 35, 36.

Patients with *WDR45* mutations have lower autophagic activity and accumulation of aberrant early autophagic structures in lymphoblastoid cell lines (LCLs) 19. These findings established a direct link between autophagy dysfunction and neurodegeneration in humans 19. Research into the pathophysiology of BPAN could provide us with new insight into the role of autophagy in iron metabolism 37, and thus further increase our knowledge of pathophysiology of NBIA disorders as a whole. Currently, the primary pathophysiological process leading to NBIA disorders is still not fully understood. Whether abnormal iron deposition is a final common pathway causing disturbed neuronal dysfunction or a “biomarker” of NBIA remains to be determined 38.

## Inheritance and Gender

Beta‐propeller protein‐associated neurodegeneration is inherited in an X‐linked dominant manner, but the vast majority of cases (91.5% of the reported cases) are singletons due to de novo mutations. Most affected individuals are female, suggesting reduced survival of male embryos carrying pathogenic *WDR45* mutations 18, 38. The broad phenotypic variability in females can be at least partly explained by mosaicism due to skewed X chromosome inactivation. Disease severity in females ranges from pronounced and early disability to asymptomatic carriers. Therefore, cases should not be assumed to be de novo in the absence of clinical signs in the mother 27.

A few male patients have been described, and most seem to have similar but more severe disease 6, 16, 20, 27. Evidence suggestive of somatic mosaicism (i.e., uneven distribution in different cells and tissues) was reported in one male patient 1 offering a potential explanation for the highly variable involvement and disease severity also seen in males. In conclusion, the broad spectrum of clinical features and severity of BPAN is believed to be due to a combination of one or more of the following: the severity of the mutation, skewed X chromosome inactivation in females, and somatic mosaicism in both sexes.

## Conclusion

We have reviewed data from 68 published cases of BPAN. Our analyses show that the clinical spectrum of BPAN is highly heterogeneous. Next‐generation, broad‐spectrum genetic analyses, such as whole‐exome sequencing, have enabled early detection of BPAN also in individuals with atypical phenotypes, rendering this disorder highly relevant for both pediatric and adult neurologists. Early diagnosis and regular clinical follow‐up of patients with BPAN are essential in order to offer proper genetic counseling to the affected families, anticipate the deterioration that occurs later in the course of the disease, and provide symptomatic therapy for the movement disorder, when appropriate. The molecular pathophysiology of BPAN is only starting to become unraveled and provides an intriguing novel link between neurodegeneration and impaired autophagy. Increased insight into the molecular mechanisms underlying BPAN will enable the design of tailored therapies for this debilitating disorder and may also provide novel understanding into the role of autophagy in brain aging and neurodegeneration.

## Authorship

KES: conceived and designed the study, collected the data, performed statistical analyses, and drafted the manuscript. IOG: collected the data and drafted the manuscript. GH: collected the data and drafted the manuscript. PMK: collected the data and drafted the manuscript. CT: received fund, conceived and designed the study, collected the data, and drafted the manuscript.

## Conflict of Interest

None declared.
